# An Impact Location Algorithm for Spacecraft Stiffened Structure Based on Posterior Possibility Correlation

**DOI:** 10.3390/s20020368

**Published:** 2020-01-09

**Authors:** Lei Qi, Zhoumo Zeng, Lichen Sun, Xiaobo Rui, Fan Fan, Guixuan Yue, Yueyang Zhao, Hao Feng

**Affiliations:** 1 State Key Laboratory of Precision Measurement Technology and Instrument, Tianjin University, Tianjin 300072, China; qilei@tju.edu.cn (L.Q.); zhmzeng@tju.edu.cn (Z.Z.); ruixiaobo@126.com (X.R.); yueguixuan@tju.edu.cn (G.Y.); 2 Beijing Institute of Spacecraft Environment Engineering, Beijing 100094, China; sunlichen-007@163.com (L.S.); zhao_yyang@126.com (Y.Z.); 3 China Academy of Space Technology, Beijing 100094, China; 13488699737@163.com

**Keywords:** impact location, acoustic emission, spacecraft, stiffened structure, posterior probability

## Abstract

In order to ensure the safety of spacecrafts in orbit, impact location is an important part of structural health monitoring systems. In this paper, an impact location algorithm based on posterior probability correlation is proposed to solve the problem, that is, the impact point in the stiffened structure of a spacecraft is difficult to locate. The algorithm combines the Gaussian cross-correlation possibility weight method and the Bayesian posterior probability method. The cross-correlation possibility weight superposition based on grids was used to reduce the dependence of the accuracy of time difference extraction. Gaussian and normalized fitting were used to compensate the reflection, modal transformation, and amplitude attenuation of a stiffened plate. The location result was further optimized by the posterior probability. The proposed algorithm can be applied to the impact source localization of complex stiffened plate structures. The experiment results showed that the average location error can be 2.57 cm with proper sensor network schemes.

## 1. Introduction

With the development of space technology and aerospace engineering, the number of spacecrafts in orbit is increasing rapidly in recent years [1,2]. Spacecrafts in orbit are subject to long-term service in the space environment. Space debris and spacecrafts with orbital heights of 200 to 800 km may collide frequently, causing damage to the spacecraft structure and threatening the safety of astronauts [3,4]. In 2013, liquid ammonia leaked out from a pipeline near the solar cell wing of the International Space Station due to debris impact. On 23 August 2018, the International Space Station was hit by space debris, causing air leak in the cabin of the Missile MS-09 orbital module [5]. Thus, the identification and location of debris impacts are prerequisites for structural integrity assessments and restoration of spacecrafts, which guarantee the safety of spacecrafts and astronauts.

Nowadays, methods used in spacecraft impact location mainly include the fiber grating method [6] and the acoustic emission method [7]. A wide detection range can be realized in the fiber grating method. However, complicated layouts limit its application in complex structures of spacecrafts. For example, NASA has arranged 36 Bragg grating sensors on a 38 × 38 cm spacecraft shell plate to locate the orbital debris impact [8]. Compared with the fiber grating method, the acoustic emission method has gained a lot of attention because of its advantages of few nodes, easy implementation, and strong adaptability [9].

At present, some traditional acoustic-emission-based impact location techniques, such as the time difference of arrival (TDOA) method, have been widely used [10,11,12]. This technique collects the TDOA of acoustic signals at different sensors, with the wave speed of a signal and the positions of sensors known and the source of an acoustic signal obtained with the use of the Triangulation method [12]. The accuracy of the TDOA method mainly depends on the accuracy of the arrival time of the signal and the accuracy of the wave speed. To make the TDOA method more accurate and suitable for more materials, many researchers have improved this traditional method. Mohd et al. [13] proposed an impact location algorithm based on continuous wavelet transform. This method improves the accuracy of signal arrival time by time–frequency analysis and obtains more accurate impact location results than the traditional fixed threshold method. Ciampa et al. [14] used continuous wavelet transform to achieve impact locations in composite materials. Sharif-Khodaei et al. [15] used the multi-layer perceptron (MLP) neural network to calculate an impact location in a composite material based on various parameters such as the arrival time and the maximum signal amplitude. Salamone et al. [16] used a macrofiber composite (MFC) sensor array to determine the propagation direction of an impact signal and achieved an impact location in a variety of materials. All of the above methods can achieve accurate impact locations in ordinary flat plates.

However, for spacecrafts, in order to ensure the sufficient structure mechanical strength, some periodic stiffened structures are usually provided on an outer surface thereof. The internal Lamb wave propagation characteristics are complicated due to reflection and transmission by a stiffener. Most of the above impact location studies are based on flat plates, which cannot be utilized for spacecraft structures directly. Aiming at this problem, Li et al. [17] proposed an adaptive energy compensation threshold filtering algorithm to achieve an accurate impact location in stiffened aluminum plates. Nevertheless, the filter band in this method needs to be reselected for different structures, and the number of sensors is small with the insufficient stability of the location results. Ebrahimkhanlou and Salamone carried out many related studies. They proposed a novel, single-sensor acoustic emission (AE) source localization algorithm [18,19], with the use of the total least squares method and a multipath model. In addition, a deep-learning-based framework to localize an acoustic emission source in plate-like structures that have complex geometric was also proposed by them [20,21]. All the methods can achieve good results in experimental conditions, while some further study can also be made in more complex conditions and materials.

In order to realize accurate and stable impact locations in stiffened spacecraft structures, this paper proposes an impact location algorithm based on posterior probability correlation. According to the impact acoustic emission signal of a sensor network, a cross-correlation Gaussian possibility weight location (CCGPWL) algorithm and a posterior probability location result optimization algorithm are used to evaluate the possibility of the occurrence of impact in each area of the test plate to obtain an impact location. The method confirms the location result by the possibility weight of the multigroup cross-correlation superposition of the sensor array and reduces the requirement for the accuracy of time information. At the same time, the influence of signal reflection and amplitude attenuation in the stiffened plate is reduced by Gaussian fitting and normalization. The effectiveness of the algorithm was confirmed by experiments on three sensor network schemes and 13 impact points.

The paper is organized as follows: The method is described in Section 2. The experiment setup is introduced in Section 3. In Section 4, the results and discussion are presented. The conclusion is in Section 5.

## 2. Method

**Figure 1:**
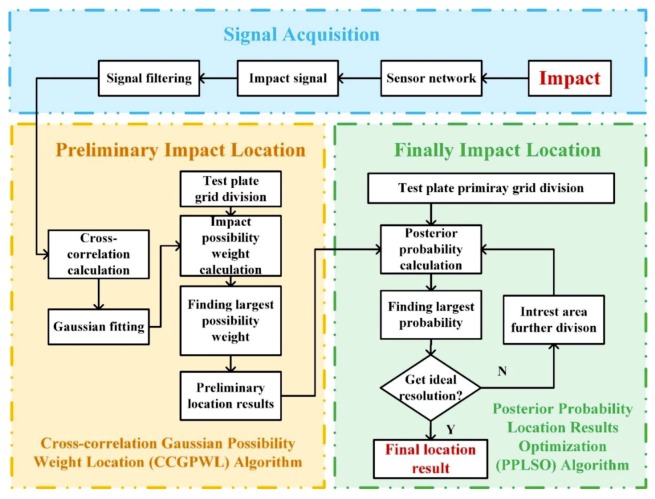
Flowchart of the posterior possibility weight correlation impact location (PPWCIL) algorithm.

In this section, the flowchart of the impact location algorithm proposed in this paper will be described in detail. As shown in Figure 1, the posterior possibility weight correlation impact location (PPWCIL) algorithm proposed in this paper can be divided into three procedures: signal acquisition, preliminary impact location, and final impact location. The signal acquisition process acquires an impact signal by a sensor network. The sensors are placed at certain places on a test plate to form the network. The preliminary impact location procedure aims to locate the impact with an acoustic emission signal directly, and the result is called a preliminary location result (PLR) in this paper. The final impact location procedure aims to reduce the location error of the PLR by processing a plurality of PLRs using a posterior probability calculation.

To obtain the PLR, the filtered signal is processed using the CCGPWL algorithm. The CCGPWL algorithm calculates the impact possibility weight of each area of the test plate with the cross-correlation curve of every two signals, and the place with the largest impact possibility weight is identified as a PLR. The CCGPWL method reduces the accuracy requirements for signal arrival time through a probabilistic model to accommodate complex signal propagation characteristics in complex stiffened panel structures. The detailed process of the CCGPWL algorithm will be described in Section 2.1.

Due to the complicated wave propagation characteristics of the stiffened structure, the PLR may have some errors. In order to reduce the location error, a posterior probability location results optimization (PPLSO) algorithm is used to get a final location result (FLR), which is more accurate. The PPLSO algorithm uses a set of PLRs as a sample to calculate the impact probability of each area with a posterior probability equation. The detailed process of the PPLSO algorithm will be described in Section 2.2.

### 2.1. CCGPWL Algorithm

As mentioned in the previous section, the CCGPWL algorithm uses cross-correlation to calculate an impact possibility weight. If there are N sensors in the sensor network and any two sensors are used as one sensor pair, *N_sp_* = (*N*^2^ − *N*)/2 sets of cross-correlation coefficients can be obtained. The cross-correlation represents the degree of correlation between two time series at different time differences. For a certain point, a delay for different paths is determined and can correspond to a value in the cross-correlation curve. The maximum value will be obtained, if the certain point is the impact location. Through the superposition of coefficients between multiple sensors, an impact possibility weight map can be obtained to determine the PLR.

**Figure 2:**
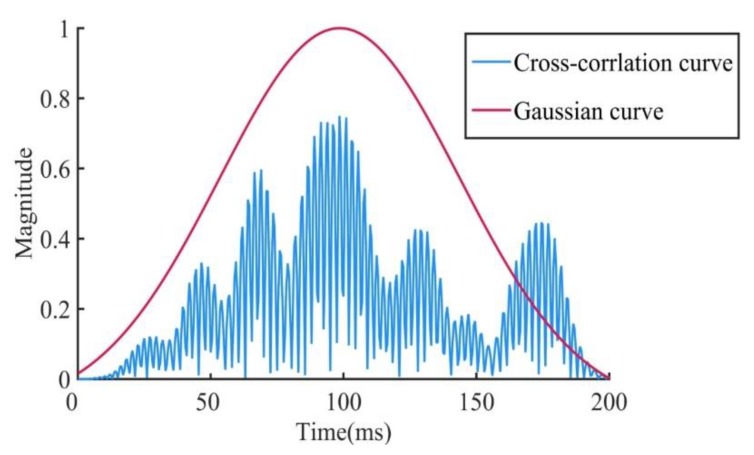
Cross-correlation curve.

Firstly, the test plate needs to be divided into small grids, in order to be able to calculate the impact possibility weight of each area of the test plate. The size of the grids is determined by the required resolution, and the grid size was 1 cm × 1 cm in this paper. After grid division, the cross-correlation curve of every two sensor’s signal is calculated. As the number of sensors used in this paper was 8, a total of 28 cross-correlation curves were obtained. In ideal situations, the curve has only one peak value. However, in situations of complex stiffened structures, since the wave produces reflection, transmission, and modal transformation, the single-peak cross-correlation characteristic becomes blurred, which may cause a location error with the CCGPWL algorithm. To solve this problem, a Gaussian fitting procedure and a normalized procedure were applied on the cross-correlation curve to obtain an ideal curve. The cross-correlation curve is shown in Figure 2 as an example. As can be seen from Figure 2, the cross-correlation curve forms a Gaussian curve that approximates a normal distribution. Such a treatment weakens the effects of reflection and refraction of the stiffener, while normalization weakens the effect of the stiffener on signal strength attenuation.

The impact possibility weight calculation method for each grid will be described below. The value of cross-correlation for one grid corresponds to the propagation distance of two sensors to the center of the grid. The impact possibility weight of each grid was calculated by Equation (1):

(1) $$I_{k}=\frac{\sum_{n=1}^{N_{sp}}P_{n}(t_{k,n})}{N_{sp}},$$

where *I_k_* is the impact possibility weight of the *k^th^* grid, *N_sp_* is the number of the cross-correlation curves, *P_n_*(*t*) is the value of the *n^th^* cross-correlation curve at *t_k,n_* and *t_k,n_* is the the time difference for two sensors at the *n^th^* sensor pair in the *k^th^* grid. If the impact is indeed located in the *k^th^* grid, the value of *P_n_*(*t_k,n_*) is the peak value of the *n^th^* cross-correlation curve, and the impact possibility weight *I_k_* is near 1 theoretically; otherwise *P_n_*(*t_k,n_*) is smaller than the peak value. In ideal conditions, the time difference for two sensors *t_k,n_* can be calculate by Equation (2):

(2) $$t_{k,n}=\frac{d_{k,n}-d_{k,n+1}}{v},$$

where *v* is the wave velocity and *d_k,n_* and *d_k,n_*_+1_ are the propagation distances of two sensors to the center of the *k^th^* grid, respectively.

After impact probabilities of all grids are calculated, the grid with the largest impact possibility weight is recognized as a PLR. Compared with the common TDOA method, the CCGPWL algorithm uses a cross-correlation curve to calculate an impact possibility weight rather than an arrival time. Moreover, the fitting and normalization of the cross-correlation curve weakens the reflection, modal transformation, and attenuation of a stiffened plate. Therefore, in principle, the CCGPWL algorithm can locate impact on a complex stiffened structure.

### 2.2. Posterior Probability Location Result Optimization Algorithm

A set of PLRs of the same impact point obtained by the CCGPWL algorithm can be expressed as *Z*, a sample vector for the PPLRO algorithm, which can be shown as:

(3) $$Z=[Z_{1},Z_{2},Z_{3}\ldots Z_{j}\ldots Z_{J}],$$

where *Z_j_* = [*x_j_ y_j_*]*^T^* is the location result vector of the *j^th^* PLR used as a PPLRO algorithm’s sample and the number of the elements in the sample vector *Z* is *J.* Due to external factors such as noise, *Z_j_* may contain certain errors, which can be expressed as:

(4) $$Z_{j}=R+e_{j},$$

where *R* is the true impact location and *e_j_* is the error contained in the PLR.

The PPLRO algorithm can optimize PLRs to obtain a more accurate and stable FLR. The PPLRO algorithm also needs to divide a test plate into small grids and locate impact location by calculating the impact probability of each grid. Different from the CCGPWL algorithm, the PPLSO algorithm uses PLRs as a sample to calculate the posterior probability of each grid and is not directly related to an impact signal. To represent the impact probability of the *i^th^* grid as *P*(*S_i_*), then *P*(*S_i_*) can be expressed as:

(5) $$P(S_{i})=\sum_{j=0}^{J}P(S_{i}|Z_{j})P(Z_{j}),$$

where *P*(*S_i_*|*Z_j_*) indicates the probability that the impact point locates within the *i^th^* grid with the sample vector being *Z*. It can be seen that *P*(*Z*), the probability that PLRs are expressed as a sample vector *Z*, does not change with the change of the calculated grid. The probability *P*(*S_i_*) of the impact location located in different grids depends on the posterior probability *P*(*S_i_*|*Z*). According to the Bayesian formula, the posterior probability equation *P*(*S_i_*|*Z*) is proportional to *P*(*Z*|*S_i_*), so the grid with the largest probability *P*(*S_i_*) is also the grid with the largest probability *P*(*Z*|*S_i_*). An FLR can be obtained by finding the maximum value of *P*(*Z*|*S_i_*).

In order to construct the probability function *P*(*Z*|*S_i_*), an event *β_j_* is introduced here. *P*(*β_j_*) represents the probability that the *j^th^* PLR in the sample vector *Z_j_* is the impact location, and *j* = 0 means that there is no PLR to accurately locate the impact location. At the same time, considering the probability that two or more PLRs accurately locate the impact location is very low, it can be assumed that:

(6) $$\sum_{j=0}^{J}P(\beta_{j})\approx 1.$$

Then, *P*(*Z*|*S_i_*) can be expanded by the full probability equation:

(7) $$P(Z|S_{i})=\sum_{j=0}^{J}P(Z|S_{i},\beta_{j})P(\beta_{j}).$$

Assuming that the occurrence of a mistake location of each PLR is independent of each other, the probability of the occurrence of a mistake location is *P_F_*. The times of the occurrence of a mistake location in the sample vector can be considered to be subject to the Poisson distribution. Then, the probability that the sample vector has *h* mistake location results can be represented as *μ_F_*(*h*). The expectation of the Poisson distribution was set to 10 in this paper, as there were 10 repeat experiments at each impact point and almost none of the PLRs achieved the right impact location. In addition, there are two cases for the calculation of *P*(*β_j_*): for *j* = 0, which means there are no correct PLRs in the sample vector and all the results are mistake location results, then the number of mistake location results is *J*, which is the number of elements in the sample vector; if *j* ≠ 0, then there is only one PLR in the sample vector, i.e., the correct location, and the number of mistake location results is *J* − 1. Then, *P*(*β_j_*) can be calculated by Equation (8):

(8) $$\left\{ \begin{array}{l} P(\beta_{j})=\mu_{F}(J),j=0\\ P(\beta_{j})=\frac{\left(1-P_{F})\cdot \mu_{F}(J-1\right)}{J},j\neq 0 \end{array}\right..$$

Assuming that the mistake location results are evenly distributed on the test plate and the number of the grids divided in the CCGPWL algorithm is *M*, then the probability that the sample vector is *Z* when the impact point locates in the *i^th^* grid and the *j^th^* PLR locates the impact point, *P*(*Z*|*S_i_*_,_ *β_j_*) can be expressed as:

(9) $$\left\{ \begin{array}{l} P(Z|S_{i},\beta_{j})=\frac{1}{M^{J}},j=0\\ P(Z|S_{i},\beta_{j})=\frac{1}{M^{J-1}}P(Z_{j}|S_{i}),j\neq 0 \end{array}\right..$$

*P*(*Z*|*S_i_*) can be expressed as:

(10) $$P(Z|S_{i})=\frac{\mu_{F}(J)}{M^{J}}+\frac{\mu_{F}(J-1)(1-P_{F})}{JM^{J-1}}\sum_{j=1}^{J}P(Z_{j}|S_{i}).$$

Assuming that the error *e_j_* obeys a two-dimensional Gaussian distribution with a mean of zero and a covariance matrix of *H*, the PLR *Z_j_* obeys a two-dimensional Gaussian distribution with a mean of *R* and a covariance matrix of *H*, which can be written as:

(11) $$P(Z_{j}|S_{i})≃Ν(R,H).$$

To simplify Equation (10), we assume that the PLR *Z_j_* obeys a one-dimensional Gaussian distribution in distance to the real impact point. Therefore, Equation (10) can be expressed as:

(12) $$P(Z|S_{i})=\frac{\mu_{F}(J)}{M^{J}}+\frac{\mu_{F}(J-1)(1-P_{F})}{JM^{J-1}}\sum_{j=1}^{J}g(d_{i,j}),$$

where *g*(*d*) is the one-dimensional Gaussian distribution, of which the variance is set to 13 by counting the variance of the PLRs of the experiments, *d_i,j_* is the distance between PLR *Z_j_* and the center of the *i^th^* grid. After calculating all the impact probabilities *P*(*Z*|*S_i_*) of all the girds, the center with the maximum impact probability is seen as the FLR.

**Figure 3:**
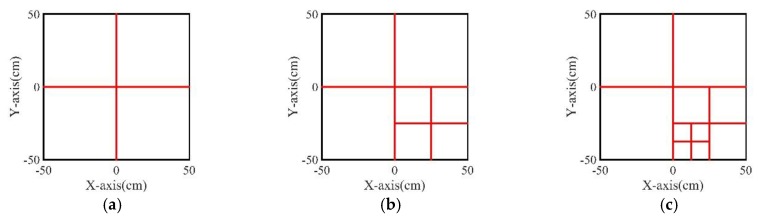
Schematic diagram of the test plate grid division: (**a**) first-stage division; (**b**) second-stage division; (**c**) third-stage division.

As it is desirable to obtain FLRs with a better resolution, the grids size may be smaller than the grids in the CCGPWL algorithm. In our experiments, the grids size for the CCGPWL algorithm was 1 cm × 1 cm, while the girds size for the PPLRO algorithm was 0.5 cm × 0.5 cm. The number of grids of the PPLRO algorithm was four times than that of the CCGPWL algorithm. In order to reduce the calculation amount, a tree search model was used in the PPLRO algorithm. As shown in Figure 3, when performing the calculation, the test plate is divided into relatively large grids for calculation firstly, and then the grid with the largest impact probability is the area of interest, which is divided into smaller grids for calculation. This process will continue, until the ideal resolution is obtained.

## 3. Experiment Platform Setup

In this section, a corresponding experimental platform was designed to verify the property of the impact location algorithm for a stiffened plate in a spacecraft. The experimental setup consisted of an acoustic emission acquisition system (DS2-16B, Softland Times, Beijing, China), eight signal amplifiers (AE Amplifier, Softland Times, Beijing, China), eight acoustic emission sensors (Nano 30, PAC, America), a test plate (material: 5A06), a computer, and a laser debris generator.

**Figure 4:**
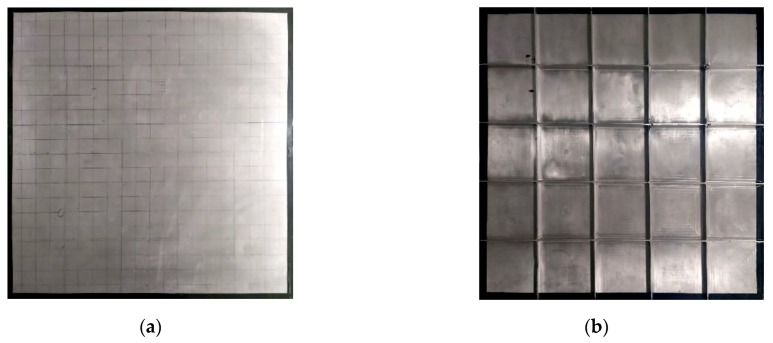
Actual structure diagrams of the test plate: (**a**) front side; (**b**) reverse side.

The test plate used in the experiment was a stiffened 5A06 aluminum alloy plate, which is commonly used in spacecraft structures. There were a total of eight stiffeners on the reverse side of the test plate. The specific parameters of the test plate are shown in Table 1. The actual structure of the test plate is shown in Figure 4.

**Figure 5:**
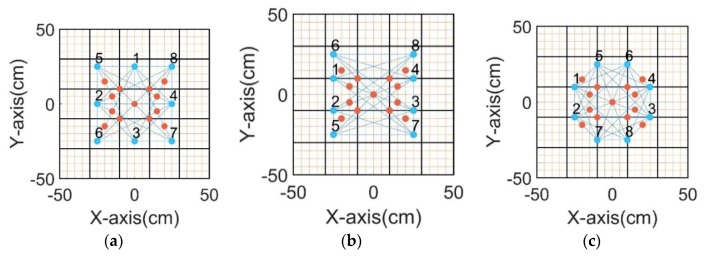
Schematic diagram of the experimental scheme: (**a**) type 1 sensor network; (**b**) type 2 sensor network; (**c**) type 3 sensor network. Blue dots indicate the sensor positions, red dots indicate the impact points, the black line at the edge indicates the boundary of the test piece, and the black line inside indicates positions of the stiffeners.

The laser debris generator mainly consisted of two parts: a laser generator and a piece of aluminum foil. The laser generator was used to generate a high-energy laser beam, and the laser beam shone on the aluminum foil and generated aluminum debris with a high speed. The speed of the aluminum debris reached 5 km/s, and its size of it was about 0.1 mm. The repetitive error of the laser debris generator was less than 0.1 mm, which met experimental requirements. During the experiment, a total of 13 impact points were selected to generate impact signals, three sensor network schemes were selected for signal acquisition, and each impact point generated impact signals 10 times. The sensor network schemes and the location of the impact point are shown in Figure 5.

**Figure 6:**
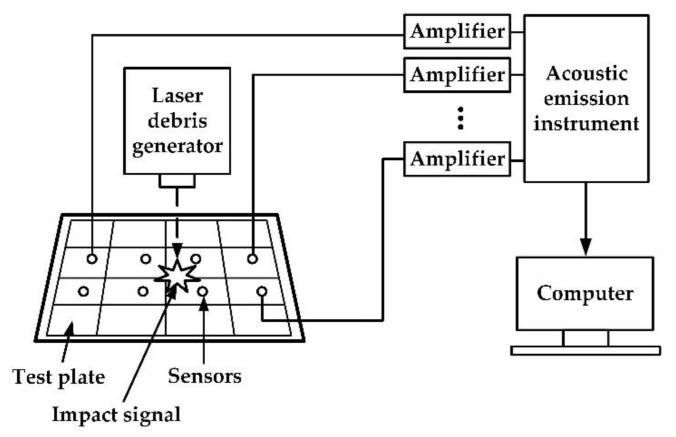
Experiment platform schematic diagram.

The experiment platform schematic diagram is shown in Figure 6. The impact signal generated by the laser debris generator was collected by the sensors. After it was amplified by an amplifier, the signal then was converted into a digital signal by an acoustic emission instrument and finally was saved in a computer. A location algorithm using Matlab codes was run and gave a location result.

Before experiments, it was necessary to calculate the speed of wave propagation in the structure, and eight Nano-30 sensors in a straight line were arranged at equal intervals. The impact signal was excited at one end of the sensor array, and the A0 mode of the signal was collected according to the eight sensors. The Lamb wave arrival time was used to calculate the velocity of a sound wave in the specimen. Taking the average of 10 measurements as the final result, the A0 mode Lamb wave velocity in the test piece was measured to be 3.12 km/s.

## 4. Results and Discussion

The data were processed with the PPWCIL algorithm proposed in this paper, and the location results were obtained. This section first explains the location process in detail through a set of data as an example. Then, the key parameters such as sensor distribution and filter frequency band are analyzed.

### 4.1. PLRs with the CCGPWL Algorithm

**Figure 7:**
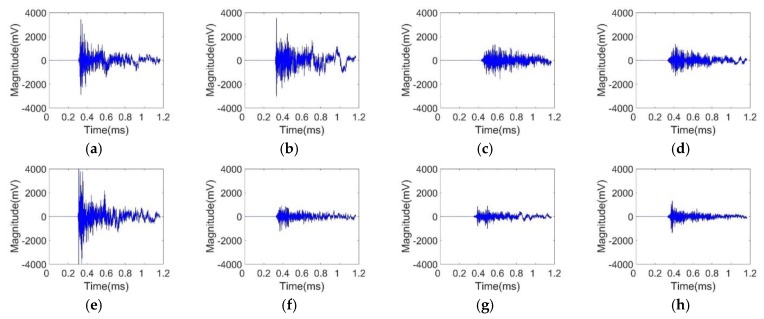
Time-domain impact signals for eight sensors: (**a**) sensor 1; (**b**) sensor 2; (**c**) sensor 3; (**d**) sensor 4; (**e**) sensor 5; (**f**) sensor 6; (**g**) sensor 7; (**h**) sensor 8.

In order to introduce the algorithm flowchart and explain the influencing factors of each process, the signals collected by the type 1 sensor network was chosen to be analyzed. The time-domain impact signals at an impact point (−20, 15) are shown in Figure 7.

From the perspective of the waveform, the time-domain map shows the characteristics of an apparent impact signal with noise. Significant spikes appeared at the beginning, and the signal gradually decreased due to the dispersion of the Lamb wave and the damping of the plate. However, due to the obvious reflection phenomenon in the stiffened plate, there was still a small-amplitude waveform after the attenuation. The method in this paper uses the cross-correlation method to calculate the impact possibility weight difference of the time difference information, it is more tolerant with the subsequently reflected signals, which made up for the insufficiency of the reflected wave intensity in the stiffened plate.

From the point of view of the arrival time, the signal obtained by the sensor near the impact point arrived earlier. From the point of view of the magnitude, the signals acquired by the sensors, which were closer to the impact point, had a relatively larger magnitude and a better curve shape. The signal propagation path corresponding to the far-distance sensor was long, and after the effect of the stiffener, the attenuation of the wave was more serious.

**Figure 8:**
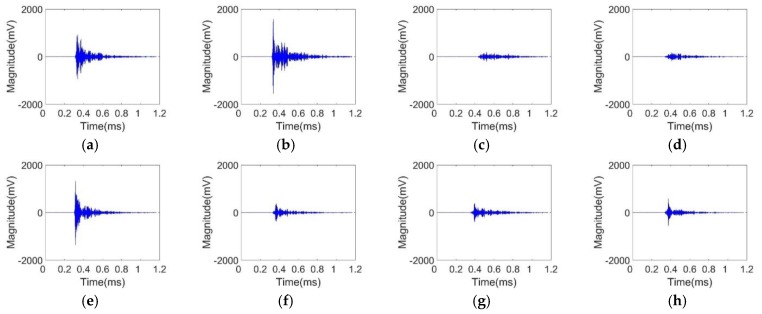
Time-domain impact signals after filtering for eight sensors: (**a**) sensor 1; (**b**) sensor 2; (**c**) sensor 3; (**d**) sensor 4; (**e**) sensor 5; (**f**) sensor 6; (**g**) sensor 7; (**h**) sensor 8.

The impact signals were subject to low-frequency ambient noise during propagation, such as noise generated by mechanical vibration. In addition, other devices generated high-frequency electromagnetic noise during operation, which also increased the difficulty of signal analysis. Based on the above analysis, for the impact source location process, the impact signal should be filtered and preprocessed first, and the low-frequency mechanical noise interference and high-frequency electromagnetic noise interference were eliminated while retaining the effective information of the signal. The filter frequency band was set as 300–400 kHz. The signals after filtering are shown in Figure 8. It can be seen that the signals had attenuation in magnitude but the quality of the signals was improved.

After obtaining the filtered signal, a cross-correlation curve and its Gaussian fitting curve were obtained. In this paper, eight sensors were used, and two sensors were selected for cross-correlation operation. A total of 28 cross-correlation curves were obtained. The impact possibility weight of each grid on the test plate was calculated according to the Gaussian curve. The difference in distance between the two sensors at the center of the grid was easy to calculate, and the time difference was obtained by the ratio between the distance and wave velocity. A time difference was used to obtain a corresponding value in the cross-correlation curve as a component of the impact possibility weight in this grid.

**Figure 9:**
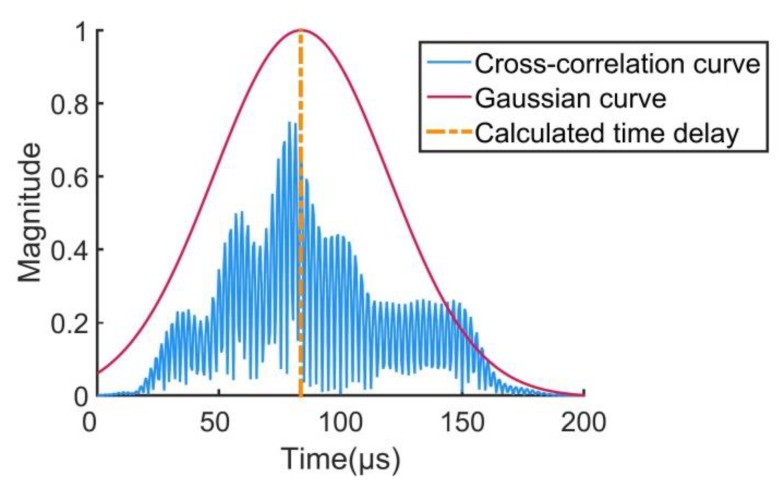
Cross-correlation curve calculated by sensor 1 and sensor 5.

In ideal conditions, the grid, in which an impact point is located, corresponds to a time delay that points to the maximum value in the Gaussian curve, as shown in Figure 9. However, the position in the time axis of the maximum value of the curve may have some offsets under the ideal conditions, called Gaussian curve offset in the paper, and will lead to a location error. In addition, the discrete error is also a reason for the location error. The discrete error is caused by the grid division. When calculating the time delay, the center of the grid is chosen as an assumed impact point, while in realistic conditions the impact location may have some distance to the grid center, causing a discrete error. The discrete error can be reduced by reducing the size of the grid but increases the amount of computation. The Gaussian curve offset is mainly caused by a low noise–signal ratio, which is usually seen in the curve calculated by signals acquired by sensors far from the impact point. By filtering the signals with a proper filter frequency band, the degree of the curve offset can be reduced.

Although some time difference errors occurred in the partial cross-correlation curve, the effect of this time error was reduced since 28 results was superimposed, which also explains the advantage of the location process by impact possibility weight in this paper and can adapt to the complex wave field effect of stiffened plates.

**Figure 10:**
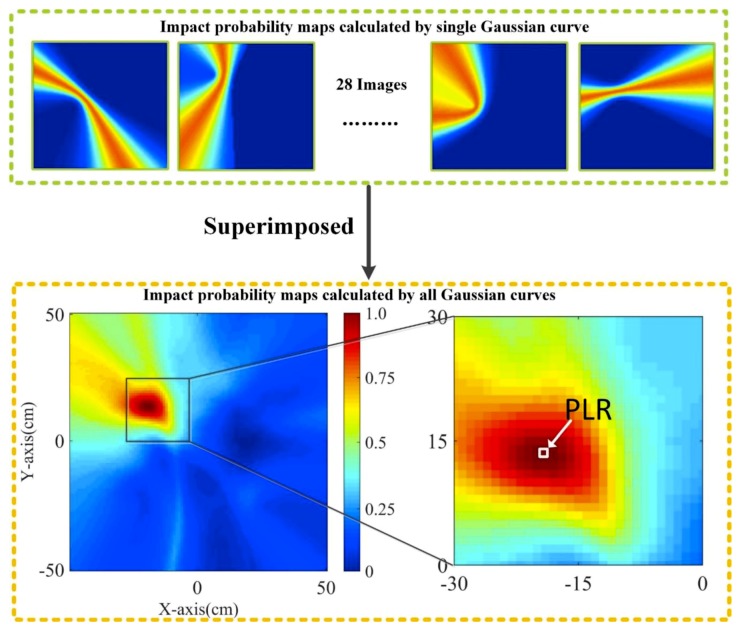
Impact possibility weight maps-drawing process.

The impact possibility weight maps-drawing process is shown in Figure 10. After combining all 28 Gaussian curves, an impact possibility weight map with a high resolution can be obtained. Each pixel in the impact possibility weight map represents the impact possibility weight *I_k_* of the corresponding grid, and *I_k_* is calculated by using Equation (1). For a set of sensor pairs, the principle of the impact possibility weight imaging for the entire region is similar to that of the TDOA method. For each grid, there is a fixed time difference that corresponds to a cross-correlated value. However, for a time difference, many points can form a hyperbola. Therefore, for a pair of sensors, the resulting curve cannot directly locate the location of the impact. In theory, two curves can get the location of the impact. The method in this paper superimposes 28 images to obtain more accurate location information. The center of the grid with the largest impact possibility weight is recognized as a PLR.

The PLRs of 10 experiments at an impact point (−20, 15) are shown as Table 2. It can be seen from Table 2 that the location results were accurate and stable. The minimal location error was 0.7071 cm and the maximum error was 1.5811 cm. The PLRs of other 12 impact points were also calculated for further processes in the next section.

### 4.2. FLRs with the PPLSO Algorithm

**Figure 11:**
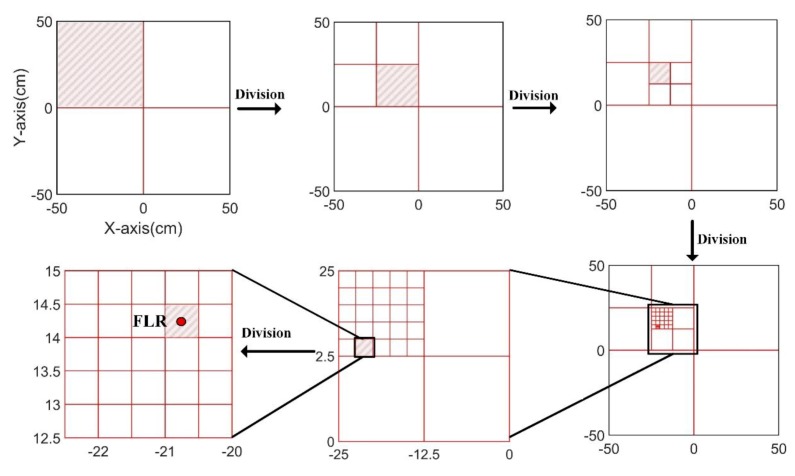
Procedure of the posterior probability location results optimization (PPLSO) algorithm.

After obtaining a set of PLRs, the PPLSO algorithm was used to obtain an FLR. The PLR of each impact point was seen as a sample vector for the PPLSO algorithm, as mentioned in Section 2.2, and each point had 10 PLRs. Take an impact point (−20, 15) as an example to introduce the PPLSO algorithm procedure. A set of PLRs obtained by the CCGPWL algorithm shown in Table 2 were used as a sample vector. The algorithm first divided the test plate into four big girds in our experiment, as shown in Figure 11. After the division, the impact probability of each grid was calculated by Equation (12). Then, the grid with the largest impact probability, as indicated by the red slash areas in Figure 11, was further divided. This procedure was repeated, until the ideal resolution was obtained. Moreover, the center of the grid with the largest impact probability in the last time division was recognized as an FLR, which was (−20.75, 14.25) for the impact point (−20, 15).

**Figure 12:**
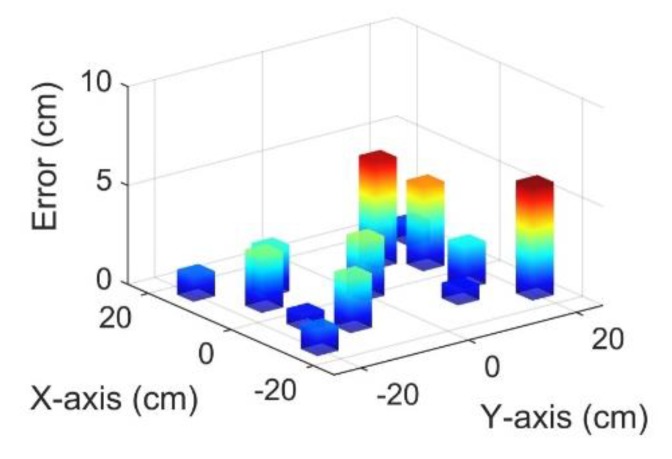
Impact probability map calculated by all Gaussian curves.

The FLR’s location error of each impact point is shown in Figure 12. The location error of FLRs was less than 6 cm with an average error of 2.57 cm. To verify the effectiveness of the PPLSO algorithm, a comparison of location errors between the average value of PLRs and the FLR of each impact point with the use of the type 1 sensor network and a filter frequency band of 300–400 kHz is shown in Table 3. It can be seen from Table 3 that, at most impact points, the location error of FLRs was smaller than that of the average value. The average error of FLRs was 2.57 cm, while the error of average value of PLRs was 4.37 cm. This means that the PPLSO algorithm can obtain a better result than the common average approach.

### 4.3. The Influence of the Filter Frequency Band and the Sensor Network Scheme on Final Location Results

**Figure 13:**
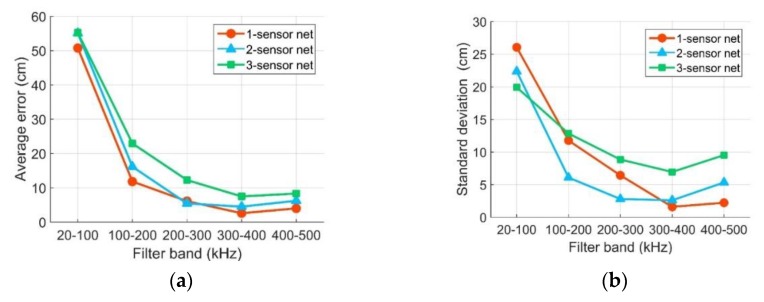
Performance comparison of the algorithm with different sensor network schemes and different filter bands: (**a**) average error curve; (**b**) standard deviation curve.

In the PPWCIL method studied in this paper, the key parameters are the filtered frequency band and the sensor network scheme. In this section, the effects of the above parameters are statistically analyzed. The final location average errors and standard deviations of the errors in different cases are shown in Figure 13. All types of sensor network had a large average error in the low-frequency filter band, and with the increase of the filter band frequency, the average error became smaller, while the optimal value was obtained at a frequency band of 300–400 kHz. The minimum average error was obtained with the type 1 sensor network in the filter frequency band of 300–400 kHz. The minimum average error was 2.57 cm. As shown in Figure 13b, the relationships between average error and filter band showed a similar trend to those in Figure 13a, and the minimum error standard deviation was also obtained with the type 1 sensor network in the filter frequency band of 300–400 kHz, of which the value was 1.59 cm. In summary, the optimal FLR can be obtained using the type 1 sensor network and the filter frequency band of 300–400 kHz. A more detailed analysis will be made below.

During the location process, the location accuracy of the CCGPWL algorithm directly affected the accuracy of the final result. Among them, whether the cross-correlation curve can obtain the maximum value in the time difference corresponding to the real impact point is the most critical. In the ideal case, the location of the peak value of the Gaussian curve accurately corresponded to the distance between the two sensors, which were used to calculate the curve. In this case, the grid, which was closer to the impact location, obtained a larger impact possibility weight. However, since the influence of the stiffeners and signal attenuation, the Gaussian curve has some offsets, which causes location errors, as mentioned in the previous section.

In the course of the experiment, the true time difference *Treal* by a true distance from the pair of sensors was calculated. In the Gaussian cross-correlation curve, the peak corresponds to a time difference *Tpeak*. If they can match exactly, they will get the best results. Therefore, the difference can be defined as:

(13) Terror=|Treal−Tpeak|.

**Figure 14:**
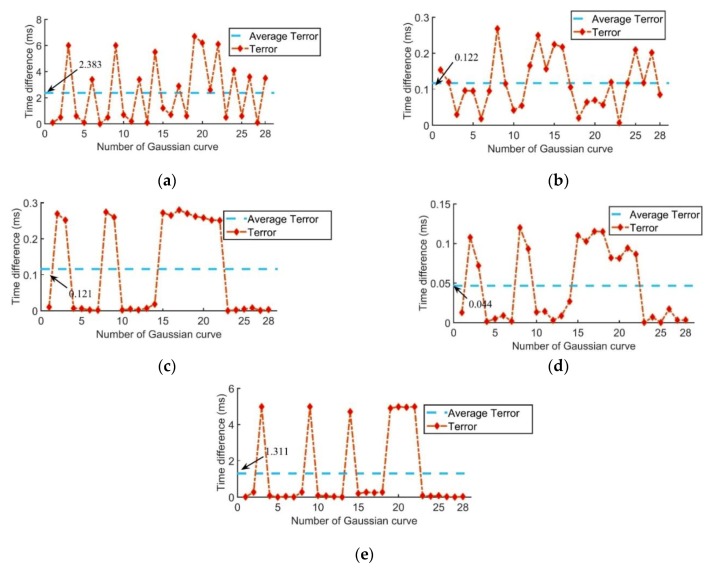
*Terror* with different filter frequency bands: (**a**) 20–100 kHz; (**b**) 100–200 kHz; (**c**) 200–300 kHz; (**d**) 300–400 kHz; (**e**) 400–500 kHz.

*Terror* is a parameter worth studying. A set of impact signals of the impact point (−20, 15) collected by the type 1 sensor network were used to compare the *Terror* with different filter frequency bands, as shown in Figure 14. The abscissa in Figure 14 represents the 28 sets of cross-correlation curves corresponding to the eight sensors used in this paper.

The average *Terror* with a frequency band of 20–100 kHz was 2.38 ms, which was larger than the other frequency bands and is the main reason leading to a large location error. The average *Terror* with a frequency band of 400–500 kHz was also relatively large, but it can be seen from Figure 14 that the number of curves with a large deviation is only six, which is much less than that in 20–100 kHz, leading to a smaller location error. Further, the best frequency band was 300–400 kHz with the smallest average *Terror* of 0.044 ms.

**Figure 15:**
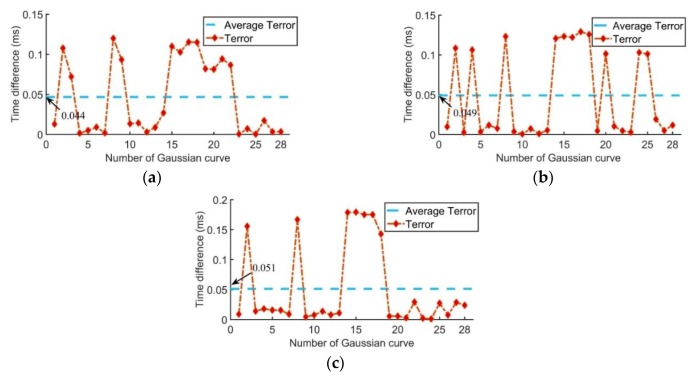
*Terror* with different sensor network schemes: (**a**) type 1 sensor network; (**b**) type 2 sensor network; (**c**) type 3 sensor network.

A similar comparison was also made with different sensor network schemes. Maintaining the filter frequency band of 300–400 kHz and using impact signals acquired by different sensor networks, the *Terror* are shown in Figure 15. It can be seen that the difference of offset with different sensor network schemes was not as obvious as that with different filter frequency bands, but it has the same trend, that is, the smaller the *Terror*, the smaller the location error.

### 4.4. Comparison between the CCGPWL Algorithm and the TDOA Method

**Figure 16:**
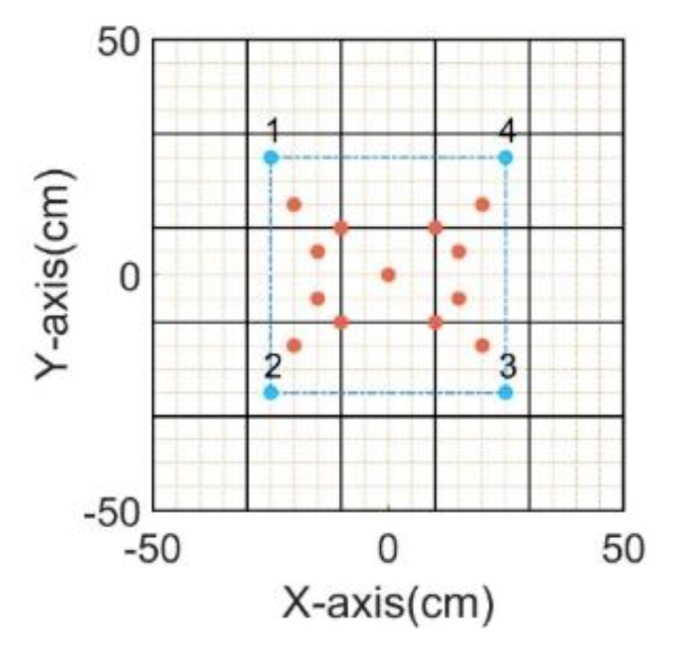
Schematic diagram of the experimental scheme. Blue dots indicate the sensor positions, red dots indicate the impact points, the black line at the edge indicates the boundary of the test piece, and the black line inside indicates positions of the stiffeners.

**Figure 17:**
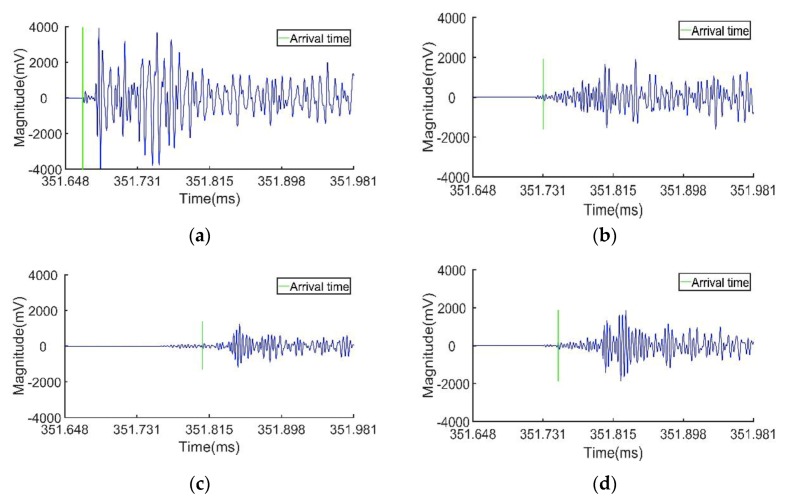
Time-domain impact signals after filtering: (**a**) sensor 1; (**b**) sensor 2; (**c**) sensor 3; (**d**) sensor 4.

To compare the performance between the CCGPWL algorithm and the TDOA method, the traditional TDOA method was used to process the same signals to obtain location results. The TDOA method in this paper uses signals acquired by four sensors. The sensors’ locations and impact points are shown in Figure 16. The time differences between sensor 1 and sensor 2, sensor 2 and sensor 3, sensor 3 and sensor 4, and sensor 4 and sensor 1 were used. In addition, the average value of all the crossover points between the hyperbola curves calculated by the time differences was regarded as the location result. The signals were filtered with a frequency band of 100–400 kHz. The threshold of the TDOA method was set to 130 mV, which caught the arrival time of the S0 Lamb wave. The wave speed of the S0 Lamb wave was calculated with an experiment. The wave speed of the S0 Lamb wave was 5.31 km/s. The arrival time obtained by signals of the impact point (−25, 15) is shown in Figure 17. It can be seen from Figure 17 that, as the paths from the impact point to the sensors are different, the signals’ attenuations are different because of the influence of the distance and stiffener. This may affect the accuracy of the arrival times obtained by using a fixed threshold and lead to a location error.

The average errors of location results calculated by the TDOA method are shown in Table 4. It can be seen from Table 4 that more than half of the impact points had bad location results with the use of the TDOA method. It was shown that the impact point, which was closer to the center of the sensor network working area, had normal location results, while those points, of which the paths to sensors and the number of passed stiffeners were very different, were no longer located with the TDOA method. This phenomenon showed that, in a stiffened plate, the position of the impact point affects the performance of TDOA method significantly. In addition, by comparing the results of the impact points, of which the TDOA average error was less than 50 cm, we can find that the average error of the TDOA method was 9.85 cm while the value of the CCGPWL algorithm was 6.37 cm. It was shown that CCGPWL algorithm performs moderately better than TDOA method. In conclusion, the accuracy of the CCGPWL algorithm is slightly better than the TDOA method, but the stability of the CCGPWL algorithm is much better than that of the TDOA method.

## 5. Conclusions

This paper proposes a posterior probability correlation impact location algorithm, which can locate impact locations in stiffened structures of spacecrafts. The accuracy and stability of this algorithm were verified by experiment, and the influences of sensor network scheme and filter band were also studied. The results showed that the proposed algorithm is adaptive to the collision location of spacecraft complex stiffened plates, and good location results can be obtained in a complex beam propagation environment of stiffened plates. The experiment results also showed the influence on the FLR of the difference in the sensor network scheme was relatively small. The posterior probability correlation impact location algorithm shows high accuracy and stability, with an average location error of 2.57 cm. The comparison between the CCGPWL algorithm and the TDOA method shows the CCGPWL algorithm has good stability while the accuracy of the CCGPWL algorithm can be further improved in future study.

## Figures and Tables

**Table 1:** Parameters of the test plate.

Parameter	Value	Parameter	Value
Length of the plate (mm)	1000	Height of the stiffener (mm)	20
Width of the plate (mm)	1000	Thickness of the stiffener (mm)	4
Thickness of the plate (mm)	2.5	Spacing of the stiffener (mm)	200

**Table 2:** Preliminary location result (PLRs) of 10 experiments at an impact point (−20, 15).

Number.	Result	Error (cm)	Number	Result	Error (cm)
1	(−19.5, 13.5)	1.5811	6	(−20.5, 14.5)	0.7071
2	(−19.5, 13.5)	1.5811	7	(−20.5, 13.5)	1.5811
3	(−20.5, 14.5)	0.7071	8	(−20.5, 15.5)	0.7071
4	(−19.5, 13.5)	1.5811	9	(−20.5, 14.5)	0.7071
5	(−20.5, 13.5)	1.5811	10	(−19.5, 13.5)	1.5811

**Table 3:** Final location results (FLRs) of 13 impact points.

Impact Location	Average Value of PLRs Errors (cm)	FLR Error (cm)	Impact Location	Average Value of PLRs Errors (cm)	FLR Error (cm)
(−20, 15)	1.00	1.06	(20, −15)	9.10	5.71
(−10, 10)	9.54	2.37	(10, −10)	7.82	0.79
(−15, 5)	8.12	2.85	(15, −5)	1.52	2.15
(0, 0)	3.58	3.02	(20, 15)	1.00	1.06
(−20, −15)	1.32	1.27	(10, 10)	5.85	5.39
(−10, −10)	2.80	2.76	(15, 5)	4.60	4.25
(−15, −5)	0.58	0.79			

**Table 4:** Comparison between the CCGPL algorithm and the time difference of arrival (TDOA) method.

Impact Location	CCGPWL Average Error (cm)	TDOA Average Error (cm)	Impact Location	CCGPWL Average Error (cm)	TDOA Average Error (cm)
(−20, 15)	1.23	55.99 (>50)	(20, −15)	12.11	137.57 (>50)
(−10, 10)	8.31	3.44	(10, −10)	9.75	4.49
(−15, 5)	14.54	148.43 (>50)	(15, −5)	3.95	12.95
(0, 0)	6.40	13.57	(20, 15)	1.15	207.53 (>50)
(−20, −15)	1.59	83.37 (>50)	(10, 10)	6.69	14.12
(−10, −10)	3.13	10.54	(15, 5)	6.13	68.13 (>50)
(−15, −5)	1.76	106.63 (>50)			
